# Visual guidance can help with the use of a robotic exoskeleton during human walking

**DOI:** 10.1038/s41598-022-07736-w

**Published:** 2022-03-10

**Authors:** Myunghee Kim, Hyeongkeun Jeong, Prakyath Kantharaju, Dongyual Yoo, Michael Jacobson, Dongbin Shin, Changsoo Han, James L. Patton

**Affiliations:** 1 Mechanical and Industrial Engineering, University of Illinois at Chicago, Chicago, IL 60607 USA; 2 Department of Robot Engineering, Hanyang University, 55 Hanyangdaehak-ro, Ansan, South Korea

**Keywords:** Biomedical engineering, Mechanical engineering

## Abstract

Walking is an important activity that supports the health-related quality of life, and for those who need assistance, robotic devices are available to help. Recent progress in wearable robots has identified the importance of customizing the assistance provided by the robot to the individual, resulting in robot adaptation to the human. However, current implementations minimize the role of human adaptation to the robot, for example, by the users modifying their movements based on the provided robot assistance. This study investigated the effect of visual feedback to guide the users in adapting their movements in response to wearable robot assistance. The visual feedback helped the users reduce their metabolic cost of walking without any changes in robot assistance in a given time. In a case with the initially metabolic expensive (IMExp) exoskeleton condition, both training methods helped reduce the metabolic cost of walking. The results suggest that visual feedback training is helpful to use the exoskeleton for various conditions. Without feedback, the training is helpful only for the IMExp exoskeleton condition. This result suggests visual feedback training can be useful to facilitate the use of non-personalized, generic assistance, where the assistance is not tuned for each user, in a relatively short time.

## Introduction

Aging, neurological diseases, and musculoskeletal disorders can result in reduced mobility in diverse populations, such as impaired gait performance. Reduced mobility is highly associated with reduced quality of life and life expectancies^1–3^. Wearable robotic devices have been proposed as a promising technology in the rehabilitation field for restoring walking performance, such as walking speed, cadence, and stride length. For instance, robotic ankle–foot devices have been intended as a potential aid for patients with peripheral or central nervous system disorders or for lower-limb amputees^4–6^. Rapid progress in technology now enables robotic exoskeletons to adapt to each user based on the user’s biofeedback signals, including energy consumption, metabolic rate^7–9^, and muscle activities^10–12^. Using a well-customized robotic exoskeleton, users are able to greatly reduce their physical effort, measured by their metabolic rate^6,9,13^. On the other hand, the user also needs to adapt to the wearable robot to obtain the best assistance benefits; however, there is no agreed upon methodology, best practice, or standards to facilitate user adaptation to the robotic exoskeleton.

When individuals use robotic devices, they need to adjust their movements as they adapt to the robot and learn how their motions change^14^. When a physical perturbation is applied (by a robot or external forces), subjects tend to provide a compensating torque that negates the new force and returns their movement to that before the perturbation^11,12,15^. Therefore, even healthy young users need time to adapt their locomotion to account for the physical change caused by the wearable robotic device^16,17^. Without such user adaptation, the use of a robotic device can impede the wearer’s attempted range of motion, which inadvertently can cause injury^18^ and perhaps reduced comfort and metabolic economy. When a user was provided sufficient time to adapt to a robot (approximately 14 min), Galle et al.^19^ observed increased metabolic economy. Often, researchers provide at least one day of training for users to acclimate to the device with simple instructions prior to evaluation^5,20^. It was also noted that after three days of training on exoskeleton use, the metabolic cost of walking could change from a 7% increase over baseline to a 10% decrease^21^. These results suggest that metabolically inefficient assistance from a wearable robot may be the result of insufficient user adaptation to the robot. Despite the importance of user adaptation to a robotic device, only a few studies have investigated efficient and effective methods for helping users adapt to a wearable robotic device in a relatively short time for a non-personalized condition^17,19,20,22–24^.

For rehabilitation training, user guidance in the form of instruction and sensory feedback (i.e., haptic, visual, auditory, etc.) is usually provided to help individuals adjust their movement patterns. After receiving instruction in motor exploration, users appeared to improve their confidence while using a robotic device, an important factor in rehabilitation^25^, and to enhance their ability to appropriately use a lower-limb wearable robot^25,26^. These previous studies, however, focused on the effect of wearable robot assistance on the user’s gait, not on a training protocol; therefore, the true effect of the instruction set for motor exploration remains unclear. On the other hand, sensory feedback has been used to promote user adaptation to robotic devices by providing visual^27–29^, auditory^25,29^, and kinesthetic/tactile perception feedback^27,29^. In particular, visual feedback appears to effectively provide information to adjust the user’s movement^26–28^ and to enhance motivation and concentration^25^. The visual feedback method was shown to have a larger impact than kinetic guidance on modifying how healthy individuals walk on a treadmill^27^. With visual feedback, users greatly improved their use of the exoskeleton to achieve the target outcome^26^. This visual feedback can be done using the tracking error information of a robot to motivate the movement of the user, resulting in improved rehabilitation outcomes^30^. It is possible that visual feedback with instruction may facilitate wearable robot use and therefore reduce walking effort after visual guidance training.

**Figure 1:**
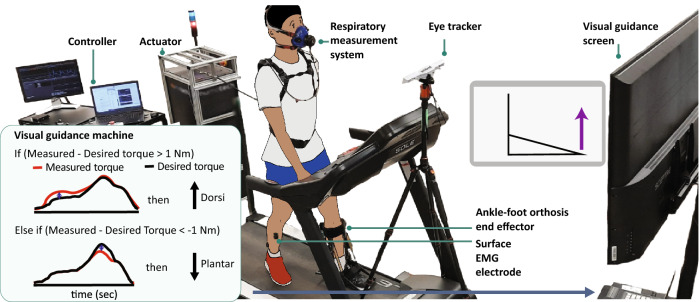
Experimental setup of visual guidance to facilitate the wearable robot use. The participant walked on a treadmill with a unilateral ankle–foot orthosis while being measured for respiratory rate, electromyography (EMG), and pupil diameter. The subject received visual guidance from software with a graphical user interface. The visual guidance presented an animation with an arrow indicating the error between the desired and actual torque. A positive “Up” and a negative “Down” arrow indicated that the user did not provide enough actual dorsiflexion torque and plantarflexion torque, respectively.

**Figure 2:**
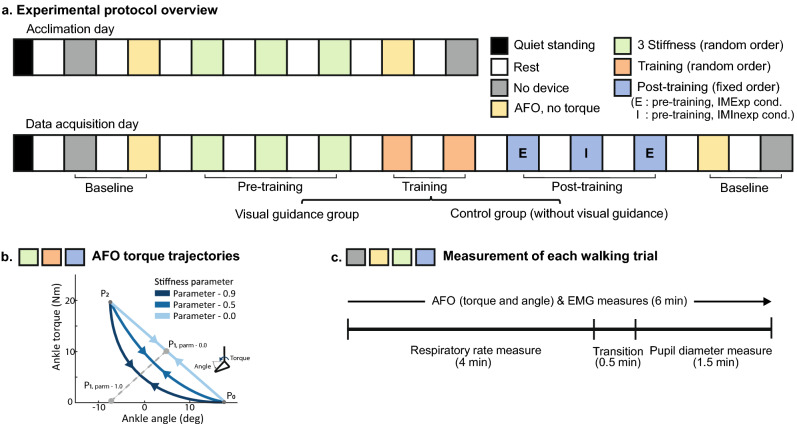
Experimental protocol. (**a**) We conducted two days of experiments; day1: acclimation to a robotic ankle exoskeleton and day2: data acquisition to investigate the effect of visual guidance on ankle exoskeleton use. (**b**) Participants experienced three different ankle exoskeleton torque trajectories as a function of a stiffness parameter on the acclimation day and data acquisition day, pre-training period. After pre-training, initial metabolic inexpensive (I), and metabolic expensive (E) exoskeleton conditions were selected and presented during training and post-training periods. (**c**) We measured respiratory rate for the initial 4 min and pupil diameter for 4.5–6 min. We measured robotic ankle exoskeleton torque and angle and electromyography (EMG) for the entire period.

In this study, we investigated the importance of user guidance training in the form of visual biofeedback with visual instruction in facilitating the use of a wearable robot, specifically a robotic ankle exoskeleton. The robotic ankle exoskeleton assists in either plantarflexion or dorsiflexion during walking^5,6^ by providing torque assistance at specific phases^5,31^. We hypothesized that visual guidance through visual feedback of robotic ankle exoskeleton use will guide the users to optimize their walking patterns to best use the robot. Specifically, we hypothesized that the initially metabolically expensive (IMExp) exoskeleton condition would result from lack of user adaptation to a robot; therefore, after the user learns to better use the robot with visual guidance for a short time frame, the user will more efficiently use the robot, as shown by reduced metabolic cost. We also hypothesized that when the robot parameter is well-tuned to reduce walking effort (the initially metabolic inexpensive (IMInexp) condition), visual guidance will help the user to continue to use the robot efficiently. We tested the hypotheses by providing visual guidance to the Visual guidance group (Fig. 1) and no visual guidance to the Control group while participants walked on a treadmill while wearing a robotic ankle exoskeleton, and we assessed the change in metabolic cost before and after robot use training. For both of the groups, we first identified subject-specific IMInexp and IMExp exoskeleton conditions as a baseline using a standard parameter search, motivated by prior personalization studies^9,32,33^. Then, we provided robot use training periods with/without visual guidance for both exoskeleton conditions of initially metabolic expensive and inexpensive ones, and finally evaluated the effect of the training on the wearable robot use (Fig. 2). In addition to the metabolic cost of walking, we looked into the changes in muscle activity, gait symmetry, and cognitive load to understand users’ movement adjustment methods.

## Results

### Physical characteristics

The independent *t*-test indicated insignificant differences in physical characteristics between the visual guidance group and the control group for age (visual guidance group: 23.7 ± 3.8 years; control group: 22.7 ± 4.6 years, *p* = 0.626), height (visual guidance group: 176 ± 8.4 cm; control group: 175.1 ± 7.6 cm, *p* = 0.795), and weight (visual guidance group: 78.2 ± 10.8 kg; control group: 78.4 ± 11.1 kg, *p* = 0.977), respectively.

### Metabolic cost

**Figure 3:**
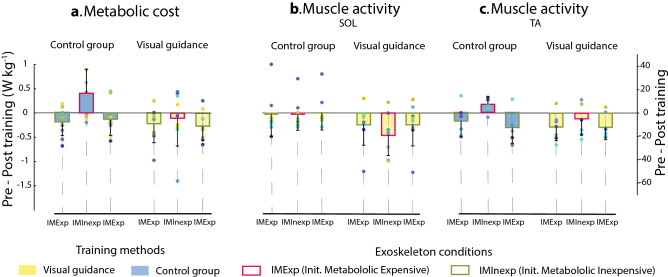
The change in (**a**) metabolic cost, (**b**) soleus and (**c**) tibialis anterior activation levels after training, compared to the pre-training (Pre-Post training). Each bar represents the mean of the difference in normalized pre-training and post-training outcomes for the training methods (Visual guidance (yellow), Control group (blue)) and the exoskeleton conditions (initially metabolic expensive (IMExp, red outline), and initially metabolic inexpensive (IMInexp, olive outline)). Error bars indicate standard deviation. Colored dots shows the individual responses for each subject.

**Figure 4:**
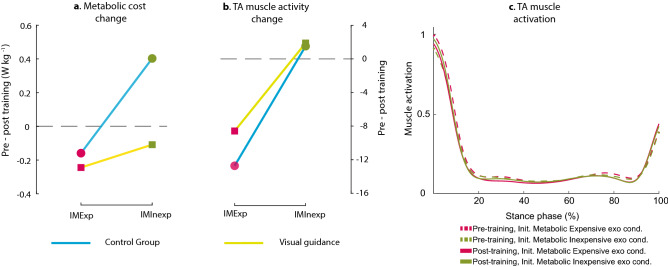
Effects of training methods (Control, and Visual guidance) and exoskeleton conditions (IMExp, IMInexp) on change in (**a**) Metabolic cost and (**b**) TA muscle activity. (**c**) TA muscle activation during the stance phase. Both training methods helped to change in metabolic cost and TA muscle activity (Pre-Post training) for the Init. Metabolic Expensive (IMExp) condition compared to the Init. Metabolic Inexpensive (IMInexp) condition. The Visual guidance helped to change in metabolic cost, compared to the Control group.

A two-way mixed-effects ANOVA revealed that there were no statistically significant interactions between the effects of training methods and exoskeleton conditions (F(1, 17) = 3.4, *p* = 0.15) (Figs. 3a, 4a). Simple main effects analysis showed that both training methods and exoskeleton conditions have a statistically significant effect on the change in metabolic cost between pre and post training (F(1, 17) = 4.8, *p* = 0.04; F(1, 17) = 6.1, *p* = 0.02, respectively). After the Visual guidance training, the participants reduced their cost of walking during the post-training compared to the pre-training conditions by 0.18 W·Kg^−1^ on average. For the control group, the participants increased the cost by 0.12 W·Kg^−1^ on average. Regardless of training methods, participants reduced the metabolic cost of walking for the IMExp condition by 0.2 W·Kg^−1^ while they increased the cost by 0.15 W·Kg^−1^ on average for the IMInexp condition. Between the two groups, the metabolic cost of walking of the averaged two unpowered conditions and the averaged two normal walking conditions were not different (independent *t*-test, *p* > 0.16).

### Muscle activation: soleus

A two-way mixed-effects ANOVA revealed that there were not statistically significant interactions between the effects of training methods and exoskeleton conditions (*p* > 0.09) (Fig. 3b). Simple main effects analysis showed that training methods and exoskeleton conditions did not have a statistically significant effect on the change in soleus muscle activity (*p* > 0.2). Between the two groups, the soleus muscle activity of the averaged two unpowered conditions was not different (independent *t*-test, *p* = 0.19).

### Muscle activation: Tibialis anterior

A two-way mixed-effects ANOVA revealed that there was not a statistically significant interaction between the effects of training methods and exoskeleton conditions (F(1, 11) = 0.3, *p* = 0.6) (Figs. 3c, 4b). Simple main effects analysis showed that the exoskeleton condition has a statistically significant effect on the change in Tibialis anterior (TA) muscle activity post-training, compared to the pre-training (F(1,11) = 12, *p* = 0.005) (Fig. 4b). Simple main effects analysis showed that the training methods did not have a statistically significant effect on the change in TA muscle activity (F(1,11) = 0.5, *p* = 0.5). There was no difference between the two groups for the tibialis anterior muscle activity of the averaged two unpowered conditions (independent *t*-test, *p* = 0.39).

Rectus femoris did not show a change in all comparisons (*p* > 0.14).

### Symmetry index

A two-way mixed-effects ANOVA showed that there were no statistically significant interaction and main effects on the change in symmetry index (*p* > 0.4).

### Torque tracking error

A two-way mixed-effects ANOVA showed that there were no statistically significant interactions between training conditions and exoskeleton conditions; and simple main effects on the change in tracking error (*p* > 0.3).

### Net mechanical energy

A two-way mixed-effects ANOVA showed that there was no statistically significant interaction between training conditions and exoskeleton conditions (*p* > 0.3). Simple main effects analysis showed that the exoskeleton conditions and training methods did not have statistically significant effects on the change in push-off work post-training compared to pre-training (*p* > 0.2).

### Cognitive load

A two-way mixed-effects ANOVA revealed that there were no statistically significant interactions between the effects of training methods and exoskeleton condition (*p* = 0.6). Simple main effects analysis showed that exoskeleton conditions and training methods did not have a statistically significant effect on the change in pupil diameter, post-training compared to pre-training (*p* > 0.1). There was no difference between the two groups for the pupil diameter of the averaged two normal walking conditions and the averaged two unpowered conditions (independent t-test, *p* = 0.099, *p* = 0.29, respectively).

## Discussion

This study investigated the effects of visual guidance training on robotic ankle exoskeleton use. We hypothesized that metabolic inefficiency during a given condition would result due to lack of user adaptation to a robot, and visual feedback with instruction would be an effective method to facilitate the user’s adaptation to the wearable robot. We also hypothesized that when assistance from a robot is helpful even with minimal human adaptation, the addition of visual feedback helps to maintain the benefits of wearable robot use. We found that deploying visual guidance to a user helped reduce the metabolic cost of walking after training regardless of the exoskeleton conditions. For the IMInexp exoskeleton condition, we found that training, with and without visual guidance, helped to reduce the metabolic cost of walking and TA muscle activity. Unlike our hypothesis, we did not find a statistically significant interaction between training methods (Visual guidance, Control group) and exoskeleton conditions (IMInexp, IMExp). This result suggests that training is helpful for the initially metabolic expensive (IMExp) exoskeleton condition, and real-time visual feedback with movement instruction is further helpful to guide the use of a wearable robot regardless of exoskeleton conditions.

Visual guidance training helped in the use of the robotic ankle exoskeleton and resulted in reduced metabolic cost after training. Visual guidance appears to contribute to correcting user movement by providing real-time visual feedback on the use of the device^26–28^, including the difference between the measured and the desired ankle torque and instruction on the desired movement direction for users to follow the desired trajectory. Therefore, the visual guidance group appeared to accurately adapt to the movement for both IMInexp and IMExp exoskeleton conditions. Unknown human–robot physical interaction influences the tracking performance of an exoskeleton. Although the torque tracking performance of an exoskeleton is mainly influenced by the hardware performance, it could also be altered by the physical human–robot interaction^34^. Wang et al., found that the tracking error could be used to provide visual feedback to a user to enhance the user’s rehabilitation outcome^30^. Previous studies also have shown the effects of virtual reality feedback in the increase of motivation during gait rehabilitation^59,60^. Similarly, in our study, the visual feedback of tracking error appears to encourage the subject’s movement^35^, helps participants learn how to interact and use the wearable robot effectively^27,36,37^ and hence reduce the metabolic cost of walking.

Both training methods helped users reduce the metabolic cost of walking and TA muscle activity for the IMExp exoskeleton condition after training, compared to IMInexp condition, and the reduction was maintained (Figs. 3c, 4c). This is similar to previous studies, which showed decreased ankle muscle activations and metabolic costs after walking training with robotic ankle exoskeletons^17,21,38^. When humans experience modifications to the environment or musculoskeletal system, the central nervous system is able to form an internal model to address the modifications and develop the model through learning^17,39^. We posited that the developed internal model might contribute to decreasing TA muscle activation and metabolic cost. Researchers found that in particular, a higher difficulty task under user's capability helps the user learn more skills^40^. In addition, a difficult task is less influenced by the prior exposure (or higher retention rate) compared to an easy task^41^. In our study, it appears that the user learned the use of IMExp exoskeleton condition during the training periods and retained the use after the IMInexp condition.

When the initial condition was already metabolically inexpensive, e.g., the IMInexp exoskeleton condition, it appears that interference from a different condition (the IMExp condition) worsens how the subject uses the wearable robot and increases the metabolic cost. Previous studies indicate that when a second motor task was learned within four hours after the completion of the first task, the motor performance for the first task was disrupted^42,43^, especially for an easier task^41^. Given that the exoskeleton conditions were separated by six-minute intervals, the IMExp condition may affect the performance of the IMInexp condition. Such interference between tasks can be reduced through visual feedback by facilitating movement switching^44^. Similarly, it appears that the visual guidance also contributed to reducing the interference. Without visual guidance, it appears that interference from a different condition, the IMExp condition, worsens how the subject uses the wearable robot and increases the metabolic cost. This increased metabolic cost for the control group suggests that the visual guidance helped to maintain the benefit of wearable robot use.

The metabolic rate can also be affected by average push-off work^45^, but it appears that this did not influence the change in metabolic rate depending on the training methods in this study. The change in push-off work was not statistically significant between training methods (*p* = 0.6) and between exoskeleton conditions (*p* = 0.2). Hence, the reduced metabolic cost after training for the visual guidance group or IMInexp exoskeleton condition can be best explained by the user adaptation to the wearable robot.

The training methods and exoskeleton conditions did not change the users’ stance time gait symmetry. It is similar to the previous findings using a unilateral ankle–foot assistance system^9,20,46^. It appears that in our study, users changed their muscle activities rather than gait symmetry.

We posited that user adaptation to robotic devices might require a cognitive process to correct the movement to motion with the robotic devices, but we did not find any statistically significant outcomes on the change in pupil diameter between pre, post trainings. Cognitive functions, mainly processed by the prefrontal cortex, contribute to motor adaptation by processing and interpreting sensory information from sensory systems and planning for revised movement based on sensory feedback^47,48^. Particularly, users may need proprioceptive information to adapt their movement to the robot’s motion, as the proprioceptive system has an important role in sensing changes in force, tension, and length of the muscles^49–52^. We assumed that after visual guidance training, participants would present increased cognitive load based on movements that were learned during the visual guidance training. As pupil diameter is a well-known indicator of cognitive load^53,54^ and the prefrontal cortex contributes to the changes in pupil diameter during a cognitive process^55,56^, we measured pupil diameter to evaluate cognitive load. Visual guidance training appeared to affect cognitive load, as shown by increased pupil diameter after training, but it was not statistically significant.

Further study is required to examine the effect of continuous adaptation to personalized assistance. Our study suggests that visual guidance can help to reduce the metabolic cost of walking after training; however, the best user adaptation method remains unclear. Previous studies suggested that when a user was presented with various conditions to identify an optimal wearable robot parameter and then had time to adapt to a near-optimal condition, the user appeared to further reduce the metabolic cost of walking^33^. Future studies can optimize the wearable robot first and then investigate the effect of visual guidance on the user adaptation without interfering with the learning process of the optimal condition and compare it with the time-based adaptation. The optimization also can be conducted to maximize an objective function that is different from the metabolic cost, such as walking speed^57^, balance-related measures^32^, or muscle activity^32^ for rehabilitation.

This study investigated the effects of visual guidance training on user outcomes for different exoskeleton conditions characterized by metabolic cost, muscle activation, torque tracking error, symmetry index, and cognitive load. Visual guidance training contributes to reducing metabolic costs. For the initially hard (IMExp) exoskeleton condition, both training methods were helpful, compared to IMInexp exoskeleton condition. Though, visual guidance appears to help reduce the metabolic cost of walking for the IMInexp condition. The results suggest that monitoring and providing visual feedback of the torque tracking error can improve the training outcomes. This result also suggests that visual guidance training can be helpful to facilitate wearable robot use, especially when assistance is not personalized (i.e., a generic condition that can reduce the metabolic cost of walking for some users while it can increase the metabolic cost for others). Future work can involve further training sessions after user adaptation to the exoskeleton, emphasizing the subject’s compliance with the torque error feedback and their performance over time. Another study could include the expansion of a training protocol with visual guidance for targeted rehabilitation outcomes.

## Methods

### Visual guidance methods to help with wearable robot use

#### Hardware platform

A cable-driven unilateral robotic ankle exoskeleton (EXO-001 Ankle Exoskeleton, Humotech, Pittsburgh, PA, USA) was utilized for this study. The ankle exoskeleton device is embedded with a tension load cell (LCM200 Miniature Threaded In-line Load cell, Futek, Irvine, CA, USA) for torque sensing purposes. The ankle exoskeleton provided active plantarflexion and passive dorsiflexion assistance during walking^18^ with maximum torque in 125 Nm. The assistance was provided using the robotic ankle exoskeleton controller, which generated ankle torque based on current ankle angle and stiffness (Fig. 2b)^58^. The stiffness was the control parameter. The controller consisted of three layers (high-, mid-, and low-level controller). In the high-level controller, the stiffness parameters were manipulated to change the ankle-angle torque curve. The mid-level controller computed the desired torque during the stance phase based on the ankle-angle torque curve. The low-level controller controlled the torque, ankle-angle position, and motor position and velocity based on desired torque and angle position from the mid-level controller. In this study, three different stiffness levels (0.0, 0.5, and 0.9 Nm/rad) were used for control parameters (Fig. 2b), which affected ankle kinetics and kinematics^59^.

#### Visual feedback with instruction

We developed visual guidance through a graphical user interface (GUI) built using MATLAB app designer, which was presented to a participant who was walking while wearing the robotic ankle exoskeleton (Fig. 1). This interface provided the most relevant visual information with the least amount of visual movement on the screen^60^. The visual guidance displayed an animation (Fig. 1) of the participant’s foot movement in the sagittal plane while walking on a treadmill with the robotic ankle exoskeleton. We added an arrow to instruct the user to perform the desired toe movement with direction and magnitude based on the tracking error between the desired and actual torque. The arrow was only displayed if the difference between the desired and actual torque was larger than 1 Nm. A positive “up” arrow indicated that the subject did not supply enough dorsiflexion relative to the desired torque, and a negative “down” arrow indicated that the subject did not supply enough plantarflexion. We expected users could use the necessary information indicated with arrows to correct their movement; therefore, the movement error would gradually decrease during continuous guidance^61^.

### Experimental methods

#### Participants

Nineteen healthy adults were randomly assigned to one of two groups: the visual guidance group (*n* = 9) and the control group (*n* = 10). Participants were excluded if they had (1) musculoskeletal disorders (e.g., muscle soreness, muscle contracture), (2) cardiorespiratory/metabolic diseases (e.g., chronic emphysema, orthostatic hypertension, cardiac arrhythmia), (3) a history of balance deficits/unexplained falls, (4) unstable medical conditions (e.g., seizures), and (5) inability to see the monitor screen in front of the treadmill without contact lenses or glasses for the visual guidance. The study protocol was approved by the University of Illinois at Chicago Institutional Review Board. All participants provided written informed consent prior to the study, which stated the picture's use after obscuring the participant's face. Informed consent was obtained for publication of Fig. 1 from the participant. All methods were conducted following the relevant guidelines and regulations.

#### Experimental protocol

Participants engaged in two days of experimental protocols that involved acclimation to the robotic ankle exoskeleton and data acquisition (Fig. 2a). The two days were separated by at least 24 h to avoid fatigue. On both days, participants first experienced a quiet standing condition. Then, they walked at 1.25 m/s^5,9,62^ on a treadmill (Sole TT8 Treadmill, Tempe, AZ, USA) with the robotic ankle exoskeleton for 6 min per condition with a 6-min break between conditions (conditions defined below). Participants also walked twice without the device (second and last trials) and walked twice in a device with a no-torque condition (third and second-to-last trials) at the same speed. The no-device condition was used to examine whether the participants experienced fatigue.

During the acclimation period (first day), all participants experienced three different robotic ankle exoskeleton stiffness conditions in a random order (Fig. 2a). During the second day, we provided visual feedback and visual instruction only to the visual guidance group. For both the visual guidance and control groups, we first identified the subject-specific metabolic inexpensive and expensive conditions, which were easy to use (decreased metabolic cost) and hard to use (increased metabolic cost), respectively. In detail, we presented three different stiffness conditions in a random order, calculated the metabolic cost of walking for each condition, and selected the metabolic inexpensive and expensive conditions. Next, we provided a training period of 6 min for each subject-specific metabolic inexpensive and expensive condition. The visual guidance group received visual feedback with instructions for adjusting their movement to minimize the error arrow. The control group was instructed to walk as naturally as possible. After the training, we evaluated the effect of the training by utilizing a single-case experimental design^20^. In this case, we presented the IMExp, IMInexp, IMExp exoskeleton conditions with a hypothesis that the inexpensive condition will be metabolically more efficient compared to the inexpensive condition. We collected muscle activity and ankle exoskeleton data for 3 min of standing and 6 min of walking. We measured the respiratory rate for 3 min standing and the initial 4 min of walking. The pupil diameter was collected for 3 min standing and the last 1.5 min of walking. While we measured pupil diameter, the participants were instructed to solve three-back tasks to prevent distraction. The 30 s between the two measurements for respiratory rate and pupil diameter were used for removing the respiration mask and preparing the three-back task (Fig. 2c).

### Data measurements and analysis

#### Metabolic cost

Metabolic energy consumption was calculated using respiratory measures (K5, Cosmed, Rome, Italy). We used the last one-minute data of volumetric intake and exhaled O_2_ and CO_2_ to calculate metabolic rate using a standard equation^63^. The steady-state metabolic cost for each condition was verified using a model-based estimation method^64^, and we also visually confirmed that the ramp-up period was not included in the steady-state metabolic cost calculation. We calculated normalized metabolic cost by subtracting the standing metabolic cost from the walking metabolic cost and then dividing the value by each participant’s body weight. We also calculated the change in metabolic cost, compared to the average unpowered conditions.

#### Muscle activities

Muscle activities were measured with wireless electromyography (EMG) at a rate of 1259 Hz (EMG, Trigno, Delsys, MA, USA). Six wireless surface EMG sensors were placed on the rectus femoris, tibialis anterior, and soleus muscles. We analyzed EMG data by applying a fourth-order bandwidth filter (10 Hz ~ 500 Hz cutoff frequency), rectifying the signals, and applying a second-order low-pass filter (10 Hz cutoff frequency). Stance phases were found for all walking conditions based on the inertial measurement units (IMU) placed on the tibialis anterior. The stance phase was the period from local minima of shank’s medio-lateral angular velocity after zero crossing as heel-strike to local minima of shank’s angular velocity before zero crossing as toe-off, which is filtered with a second-order low-pass filter (20 Hz cutoff frequency)^65–67^. The filtered EMG signals of each no-device condition during stance phases were averaged, and a peak value from each averaged signal was found and averaged. The filtered EMG signals of each walking condition during the stance phases were normalized based on the averaged peak value from two no-device conditions. Additionally, the change in EMG activity compared to the average unpowered conditions was calculated.

#### Symmetry index

Stance phase of both leg were detected by using 2 local minima of each shank’s angular velocity in medio-lateral axis after applying a second order low-pass filter with 20 Hz cutoff frequency^65–67^. Stance time symmetry index (SI) were calculated with the Eq. (1).^68^

1 $$SI=2\times \frac{{T}_{exo}-{T}_{none-exo}}{{T}_{exo}+{T}_{none-exo}}$$

where, ${T}_{exo}$ is stance time on the exoskeleton side, and ${T}_{none-exo}$ is stance time on the non-exoskeleton side.

#### Cognitive load

The cognitive load was approximated using pupil diameter from the eye tracker (GP3 Eye tracker, Gazepoint, Vancouver, BC, CA). We removed outliers of pupil diameter data for each walking condition, where outliers were defined as a value more than three standard deviations from the mean of the data. We took an average of the last one-minute pupil diameter of the left and right eye^69^. Additionally, pupil diameter data were compared with the mean pupil diameter of two unpowered conditions to calculate the percentage change in pupil diameter.

#### Torque tracking performance

The mean of torque tracking error was calculated between the desired and actual ankle torque for every 200 ms during the stance phase based on our pilot study. The mid-level controller computed the desired torque by interpolating the measured angle from an optical encoder into the ankle-angle torque curve (Fig. 2b, Eq. (2))^70^ during the stance phase when the measured ankle torque was larger than the torque threshold (5Nm).

2 $$Torque\,curve={\left(1-t\right)}^{2}{P}_{0}+2t\left(1-t\right){P}_{1}+{t}^{2}{P}_{2}$$

where $0\le t\le 1$, ${P}_{0}$ is a heel strike point, which is starting angle of the dorsiflexion phase at the torque threshold (5Nm), ${P}_{2}$ is a full dorsi-flexed point, which is the peak angle of the dorsiflexion phase when peak torque is achieved, and ${P}_{1}$ is a point varied by stiffness parameter for generating the Bezier curve.

The measured ankle torque was obtained using the data from the load cell and optical encoder in the robotic ankle exoskeleton using the Eq. (3)^70^.

3 $${\tau }_{ankle}={F}_{loadcell}\cdot {L}_{6}\mathit{sin}({a}_{5})$$

where, ${F}_{loadcell}$ is the vertical force estimated by the loadcell's output voltage multiplied by an offset gain, ${L}_{6}$ is the hypotenuse of the horizontal and vertical distances of the composite spring, and ${a}_{5}$ is the angle between the rope and the axis perpendicular to the composite spring.

#### Net push-off energy

Mechanical energy of the device was calculated by integrating the power input to the device over time during the stance phase. The power input was calculated by taking the discrete derivative of the ankle angle to obtain the ankle velocity and then taking the product of the ankle velocity with ankle torque. Ankle velocity and ankle torque data were also discretized into the stance phase by setting a threshold of approximately 5 Nm for ankle torque. After applying a threshold, a second-order Butterworth filter with a 15 Hz cutoff frequency was used to filter signal noise.

#### Statistical analysis

Statistical analyses were performed using IBM SPSS Statistics (version 27) and MATLAB. For each visual guidance group and control group, we confirmed normality using the Jarque–Bera test^5,71^ (the normal distribution of the differences in the two related dependent variables). Then, we conducted a mixed effect, two-way ANOVA (between-subject factor: training methods (visual feedback, control groups), within-subject factor: exoskeleton conditions (initially metabolic expensive (IMExp), initially metabolic inexpensive (IMInexp)) to test the effect of training methods, exoskeleton conditions on the change in user outcomes (difference between the pre-training and post-training). For the IMExp exoskeleton condition, the averaged two post-training conditions were used^20,72^ after confirming equal distribution using the Kolmogorov–Smirnov test.

We also performed the normality test for an independent *t*-test by confirming the normal distribution for each group of independent variables. When data normality was confirmed, we performed an independent *t*-test to compare the two groups’ physical characteristics (i.e., age, height, and weight). If normality was not met, we used nonparametric methods, the Wilcoxon tests (an alternative method of the paired *t*-test), and the Mann–Whitney U test (a nonparametric method of a balanced independent *t*-test).

EMG data (one participant for the visual guidance group and two participants for the control group), torque tracking error and mechanical energy data (one participant for the control group), and pupil diameter data (two participants for the visual guidance group) were not collected due to measurement device failure. Also, for tibialis anterior EMG data, we excluded one participant’s data out of eight participants (the visual guidance group) and two participants’ data out of eight participants (the control group) due to outliers confirmed using a MATLAB isoutlier function. The significance levels for all statistical analyses were defined at a *p* < 0.05.

## Data Availability

The datasets generated during and/or analyzed during the current study are available from the corresponding author on reasonable request.
